# Astrocytes Downregulate Inflammation in Lipopolysaccharide-Induced Acute Respiratory Distress Syndrome: Applicability to COVID-19

**DOI:** 10.3389/fmed.2021.740071

**Published:** 2021-10-29

**Authors:** Michal Izrael, Kfir Molakandov, Ariel Revel, Shalom Guy Slutsky, Tehila Sonnenfeld, Julia Miriam Weiss, Michel Revel

**Affiliations:** ^1^Research and Development Department at Kadimastem Ltd, Nes-Ziona, Israel; ^2^Department of Molecular Genetics, Weizmann Institute of Science, Rehovot, Israel

**Keywords:** ARDS, astrocytes, immune-modulation, inflammation, embryonic stem cells

## Abstract

**Background:** An acute respiratory distress syndrome (ARDS) is caused by the increased amounts of pro-inflammatory cytokines and neutrophil-mediated tissue injury. To date, there is no effective treatment for the ARDS available, while the need for one is growing due to the most severe complications of the current coronavirus disease-2019 (COVID-19) pandemic. The human astrocytes (AstroRx) have shown immunomodulatory properties in the central nervous system (CNS). This study aimed to evaluate the capacity of astrocytes to decrease lung inflammation and to be applied as a treatment therapy in ARDS.

**Methods:** First, we assessed the ability of clinical-grade AstroRx to suppress T-cell proliferation in a mixed lymphocyte reaction test. Next, we tested the therapeutical potential of AstroRx cells in a lipopolysaccharide (LPS)-based ARDS mouse model by injecting AstroRx intravenously (i.v). We determined the degree of lung injury by using a severity scoring scale of 0–2, based on the American Thoracic Society. The scoring measured the presence of neutrophils, fibrin deposits, and the thickening of alveolar walls. The state of inflammation was further assessed by quantifying the immune-cell infiltration to the bronchoalveolar lavage fluid (BALF) and by the presence of proinflammatory cytokines and chemokines in the BALF and serum.

**Results:** We detected that AstroRx cells were capable to suppress T-cell proliferation *in vitro* after exposure to the mitogen concanavalin A (ConA). *In vivo*, AstroRx cells were able to lower the degree of lung injury in LPS-treated animals compared with the sham injected animals (*P* = 0.039). In this study, 30% of AstroRx treated mice showed no lung lesions (responder mice), these mice presented a steady number of eosinophils, T cells, and neutrophils comparable with the level of naïve control mice. The inflammatory cytokines and chemokines, such as TNFα, IL1b, IL-6, and CXCL1, were also kept in check in responder AstroRx-treated mice and were not upregulated as in the sham-injected mice (*P* < 0.05). As a result, the LPS-treated ARDS mice had a higher survival rate when they were treated with AstroRx.

**Conclusions:** Our results demonstrate that the immunosuppressive activity of AstroRx cells support the application of AstroRx cells as a cell therapy treatment for ARDS. The immunoregulatory activity may also be a part of the mechanism of action of AstroRx reported in the amyotrophic lateral sclerosis (ALS) neurodegenerative disease.

## Introduction

An acute respiratory distress syndrome (ARDS) is a form of progressive hypoxemia respiratory failure and pulmonary edema in the absence of heart failure (1). The etiology of ARDS varies (e.g., pneumonia, sepsis, or acute pancreatitis) and great efforts have been made in intensive care medicine, but the overall mortality is still high (2, 3). Recently, a significant proportion of patients infected with coronavirus disease-2019 (COVID-19) developed viral pneumonia that caused an acute lung injury (ALI) capable of rapid progression to viral sepsis and ARDS with a high fatality rate especially in older and comorbidities populations (4–6). The phases in the development of ARDS include the exudation stage characterized by the inflammatory cell infiltration and pulmonary edema (stage 1), the proliferation of myofibroblasts (stage 2), and extracellular matrix (ECM) over-deposited (stage 3) (7). The fibrosis process (stages 2 and 3) is rapid and occurs within 1 week (8, 9). This inflammatory response process referred to as cytokine storm or cytokine release syndrome (CRS), contributes to the development of ARDS and often irreversible multi-organ dysfunction syndrome (MODS) associated with the severe-critical forms of COVID-19 (10, 11). Another key process in the development of ARDS is neutrophil accumulation in high abundance in the pulmonary microcirculation, lung interstitium, and alveolar airspace of the patients with ARDS (12). In addition, an ARDS is associated with systemic neutrophil priming, delayed neutrophil apoptosis, and clearance of neutrophils from the lungs. In animal models, lung injury could be ameliorated by reducing the number of circulating neutrophils (13).

Current management for COVID-19 (severe acute respiratory syndrome-coronavirus 2, SARS-CoV-2) infected patients with severe pneumonia and ARDS remains supportive, such as the use of anti-infection drugs, intubated ventilator-assisted breathing therapy, and extracorporeal membrane oxygenation (ECMO) (14–16). Since ARDS is associated with high mortality and morbidity, it is vital to develop new effective therapeutic approaches capable of immunomodulating the immune system and inflammatory response. This could be of great benefit in preventing the disease progression and reducing the case mortality rate in the high-risk patients with COVID-19. A growing body of evidence has shown that cell-based therapies hold therapeutic effects for ARDS. Most of the studies have focused on the therapeutic effects of mesenchymal stem cells (MSCs) (17, 18) and some studies have also investigated the possible applications of other cell types, such as pulmonary epithelial progenitors (19, 20).

Another approach using astrocytes is presented as they are the most abundant glial cell in the central nervous system (CNS). Astrocytes regulate the concentration of different neurotransmitters and ions, supply various metabolites and energy, regulate osmolarity, modulate synaptic activity, secrete neurotrophic and neuroprotective factors, promote neurogenesis (21–23), and remyelination (24). Moreover, the astrocytes are essential players in immune-modulation (25, 26). Astrocytes have a dual role as key immune modulators of the immune response of the CNS to infections, neurodegenerative disorders, and injuries (27). Following injury and in disease, their ability to respond to, and commence initial responses to injury/disease is increasingly apparent. Astrocytes serve as a contact between the CNS and the peripheral immune system. In many pathological conditions, astrocytes either secrete anti-inflammatory or pro-inflammatory factors which modulate the immune system (28–30). Astrocytes, take part in both the recruitment and restriction of leukocytes in the CNS (31, 32). Neuroinflammation is increasingly recognized as an important mediator of disease progression in patients with amyotrophic lateral sclerosis (ALS), and similar to ARDS, it is characterized by reactive tissue and infiltrating peripheral monocytes and lymphocytes (33). The astrocytes derived from the pluripotent stem cells (AstroRx) demonstrated neuroprotective A2-type characteristics, such as glutamate uptake capabilities, secretion of neuroprotective factors, promotion of axon outgrowth, and protection of motor neurons (MNs) from oxidative stress (34). Intrathecal injection of astrocytes (AstroRx) in an ALS animal model demonstrated a therapeutic benefit (34) and is being tested first in the human clinical trial in the patients with ALS (clinicaltrial.gov ID NCT03482050). The immune-modulatory effect of astrocytes outside the CNS, to the best of our knowledge, is not reported yet and can shed more light on its anti-inflammatory activity in ALS disease.

In this current study, the effect of astrocytes on ARDS is investigated for the first time. We deciphered the immunomodulatory effect of AstroRx first *in vitro* by evaluating T-cell proliferation in a mixed lymphocyte reaction test. Then, we assessed the effect of intravenous (i.v.) AstroRx injection on lung injury, lung inflammation, and survival in a lipopolysaccharide (LPS)-based ARDS mouse model. Altogether our results indicate that AstroRx has the potential to maintain immune homeostasis in the lung and thereby increase the chance of survival.

## Results

### Suppression of T-cell Proliferation by AstroRx Cells

**Figure 1:**
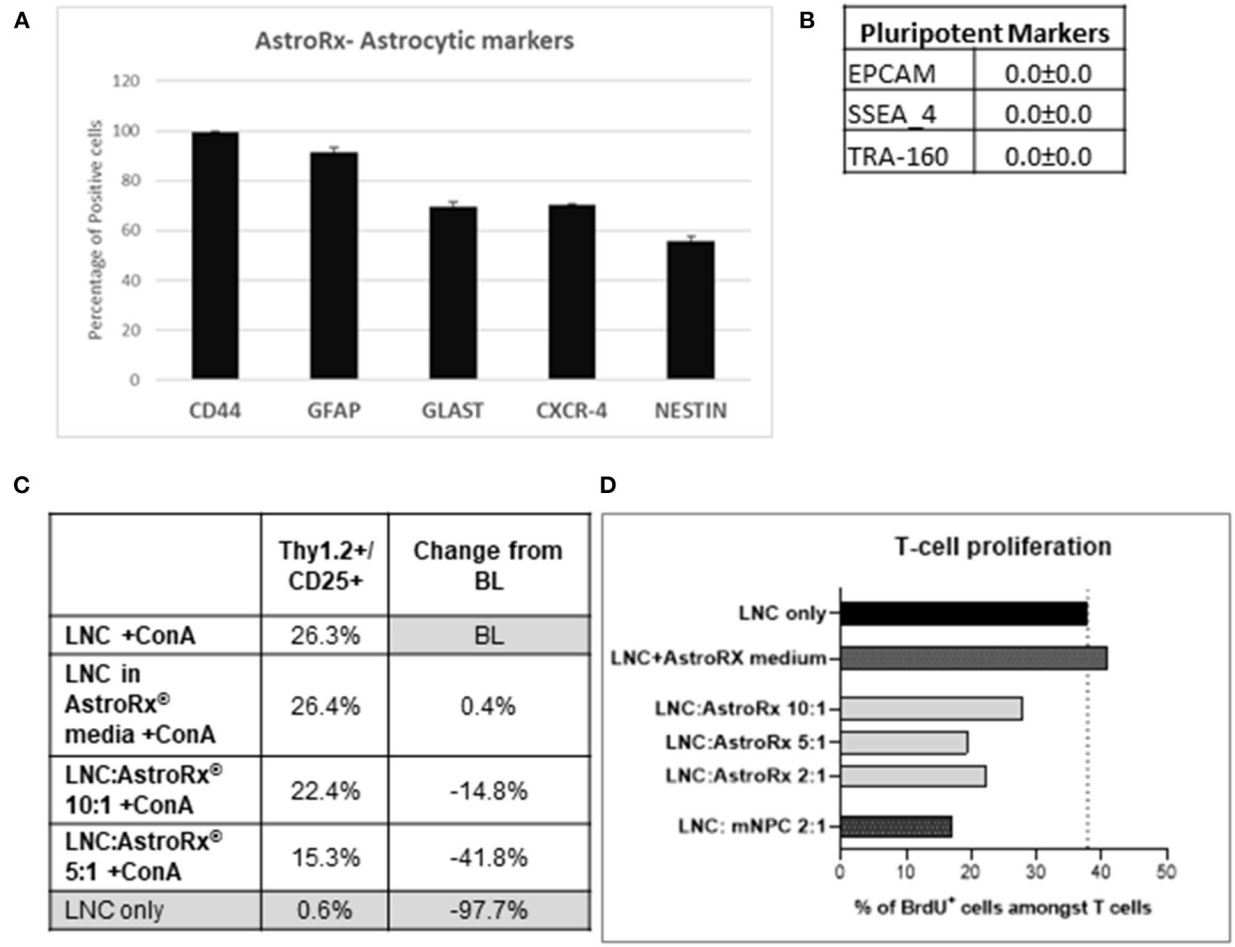
Human astrocytes (AstroRx) demonstrate immunosuppressive capacities *in vitro*. **(A,B)** The AstroRx were analyzed by flow cytometry for astrocytic markers **(A)** and pluripotent stem cells markers **(B)**. **(C,D)** The immunosuppressive potential of AstroRx was assessed in a mixed lymphocyte reaction test. The AstroRx were co-cultured with murine lymph node cells (LNCs). **(C)** The LNCs were stained for T cell marker Thy1.2 and CD25 positive cells under ConA activation in the presence or absence of different amounts of AstroRx. **(D)** The proliferation state of T cells (CD3+) was determined by the proliferation marker BrdU in the presence of ConA. The LNCs only were used as negative control while the LNC with mouse neural precursor cells (mNPC) were used as a positive control. Average and ± SEM.

In a recent study, we have shown how human embryonic stem cell (hESC)-derived astrocytes (AstroRx) protect MNs *in vitro* and in the ALS animal models (34). In these studies, we demonstrated that AstroRx protected neurons by the secretion of neuroprotective and neurotrophic factors, uptake of glutamate, and regulation of oxidative stress. The immunomodulatory capacity of these human astrocytes (AstroRx) was not fully defined yet. To better understand if AstroRx can protect from inflammatory damage, we analyzed its immunosuppressive potential. For this, we used fully differentiated AstroRx, which expressed high levels of astrocytic markers (Figure 1A) and did not express pluripotent stem cells markers (Figure 1B). The AstroRx were co-cultured with murine lymph-node cells (LNC). The T-cell proliferation in this culture was induced by concanavalin A (ConA). When ConA was not added to the culture, the percentage of T cells in the culture was below 1%. The addition of either AstroRx cells or the conditioned medium of AstroRx cells did not affect the percentage of T cells in the culture (not shown).

In contrast, in the presence of ConA, the AstroRx cells profoundly influenced the T-cell population (Thy1.2^+^/CD25^+^), which decreased by about 15% in the LNC:AstroRx® (at a ratio of 10:1) co-culture. A greater inhibitory effect was achieved when using a ratio of 5:1 of LNC:AstroRx culture, which decreased T-cell population by 42%. This effect was not observed when only AstroRx-conditioned medium was added (Figure 1C), indicating that the effect was cell-contact dependent.

To evaluate the proliferation capacity of T cells in the presence of AstroRx, we performed a BrdU cell proliferation assay. The percentage of CD3^+^/BrdU^+^ cells in a culture of LNC with ConA but without AstroRx was chosen to serve as a baseline (BL). Without ConA, the percentage of human proliferating cells among the T cells was below 1%, the addition of different amounts of AstroRx cells to LNCs in the absence of ConA did not affect the percentage of proliferating T cells (not shown). In the presence of ConA, 40% of T cells were BrdU+ and proliferating. As a positive inhibitory control, we used mouse neural precursor cells (mNPCs), which were previously described for reducing T-cell proliferation (35). Co-culture with mNPCs had no effect on T-cell proliferation in the absence of ConA (not shown) but reduced the number of proliferating cells among the T cells by more than a half when T cells were exposed to ConA (Figure 1D). Interestingly, when T cells were co-cultured with AstroRx, the proliferation rate among the T cells decreased by more than a third in the LNC:AstroRx® 10:1 culture. Higher ratios of AstroRx® to LNC resulted in a greater inhibitory effect, similar to the range of co-cultures with mNPCs (decrease by 49 or 43% in LNC:AstroRx® 5:1 or LNC:AstroRx® 2:1 cultures, respectively).

The effect was not observed when AstroRx® conditioned media was added to the culture, again suggesting that the AstroRx suppression is mediated by a direct cell- contact or communication between the AstroRx cells and LNC. Altogether, these results show that AstroRx has the potential to suppress the T-cell proliferation in a dose-dependent manner.

### AstroRx Cells Alleviate LPS-Induced Pulmonary Inflammation and Fibrosis

**Figure 2:**
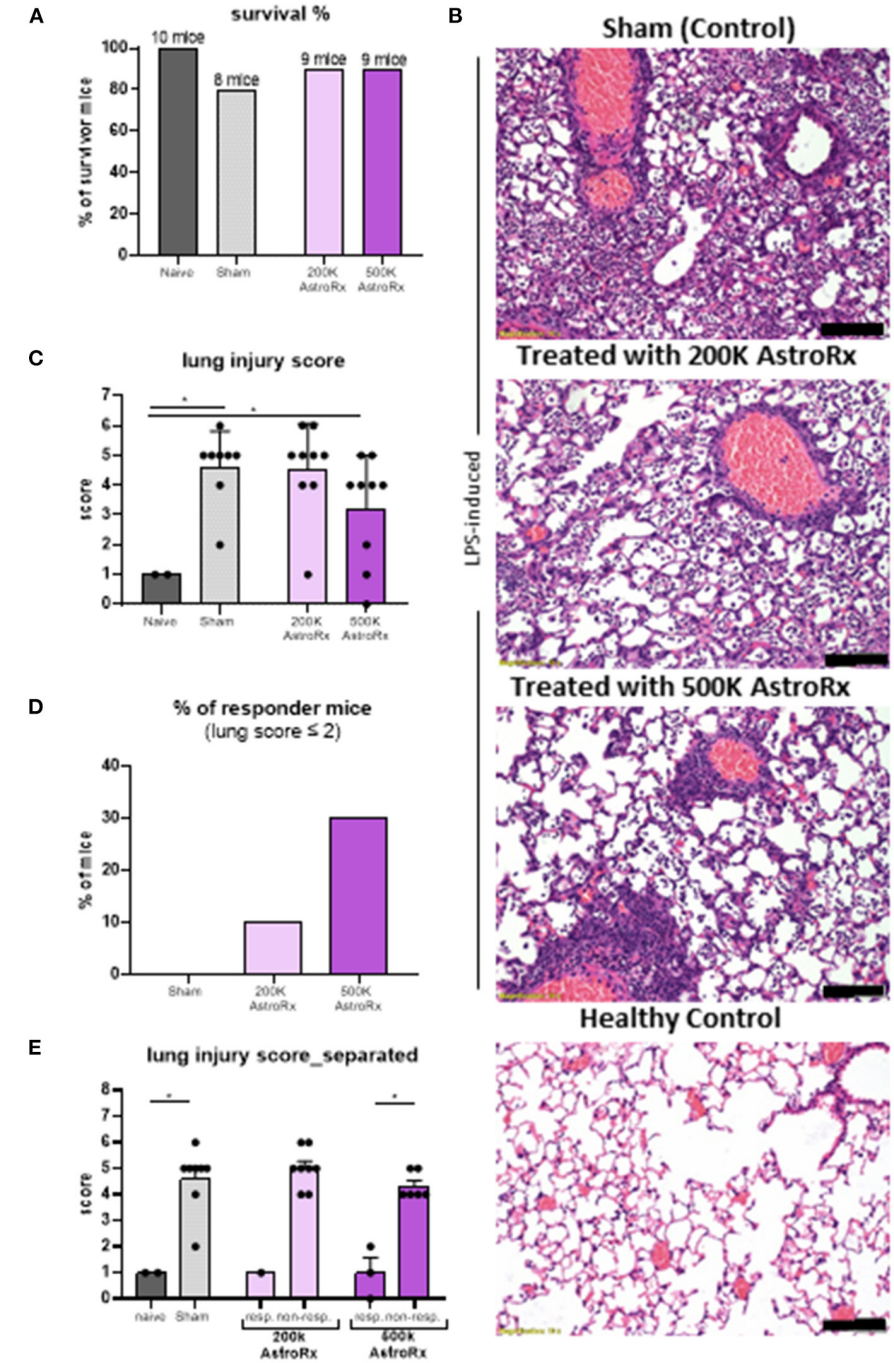
The AstroRx cells alleviate lipopolysaccharide (LPS)-induced pulmonary inflammation and fibrosis. **(A)** The survival rate of animals treated with Plasmalyte (sham) and AstroRx cells (200K or 500K per animal). **(B)** H&E staining of lung sections from top to bottom: LPS-induced treated with vehicle (PlasmaLyte), LPS-induced treated with 200K AstroRx cells, LPS-induced treated with 500K AstroRx cells, or healthy controls (no-LPS). **(C)** An analysis for acute lung injury (ALI) was performed using a severity scoring scale of 0–2 (20 fields per animal were analyzed), based on the American Thoracic Society Documents, 2011 (37). **(D,E)** According to the lung score, the mice were categorized into responders (lung score ≤2) and non-responders (lung score >2), also considering the mice that had died from ARDS (*n* = 10), the results are expressed as mean ± SEM. Mann–Whitney comparison test. **(C,D)** Naïve vs. sham: *p = 0.0222, **(C)** sham vs. 500k: *p = 0.0309, **(E)** 500k responders vs. non-responders: *p = 0.0119, *p < 0.05. Scale bar: 100 μM.

Next, we tested the immune suppressive features of AstroRx in an *in vivo* model for ARDS. Intratracheal administration of LPS in the BALB/c mice induces severe lung damage and is a prevalent ARDS animal model (36). To test whether the AstroRx was capable of limiting the lung inflammation and lung damage, we injected i.v. two different doses of AstroRx after LPS-induction (*n* = 10, 2 × 10^5^ and *n* = 10, 5 × 10^5^ cells). Naïve (*n* = 4), untreated mice and sham-injected LPS-treated mice (*n* = 10) served as the negative and positive controls, respectively. The treatment with AstroRx at both cell concentrations increased the survival of LPS-induced mice compared with the sham-injected animals (20% loss vs. 10% loss, Figure 2A). To assess the degree of lung injury in the mice that survived, we performed the histological analysis of the lung sections 72 h after an LPS treatment (Figure 2B). The sham-injected mice showed significant thickened alveolar walls and cell infiltration, while treatment with AstroRx cells at a concentration of 5 × 10^5^ cells alleviated the LPS-induced damage. An analysis of ALI was performed according to a method described by Matute-Bello et al. using a severity scoring scale of 0–2, based on the American Thoracic Society Documents (37) assessing the alveolar wall thickness, fibrin presence, and neutrophil accumulation which sums together to a total severity score (Figure 2C). The treatment with AstroRx cells significantly lowered the total severity score as compared with the sham-injected ARDS animals with 3.22 compared with 4.6 (Figure 2C, *P* = 0.039). According to lung score, the animals within one group were categorized into the responders (lung score ≤2) and non-responders (lung score >2). The percentage of responders was higher among the AstroRx-treated mice compared with the sham injected mice (Figures 2D,E), and higher among the mice that received a high dose of AstroRx compared with the mice that received a low number of AstroRx.

### AstroRx Attenuates LPS-Induced Cytokine and Chemokine Storm in Bronchoalveolar Lavage Fluid and Serum

To understand the factors that contributed to the reduced lung damage following the AstroRx treatment, we analyzed the infiltration of B and T lymphocytes, eosinophils, neutrophils, and macrophages into the bronchoalveolar lavage fluid (BALF).

**Figure 3:**
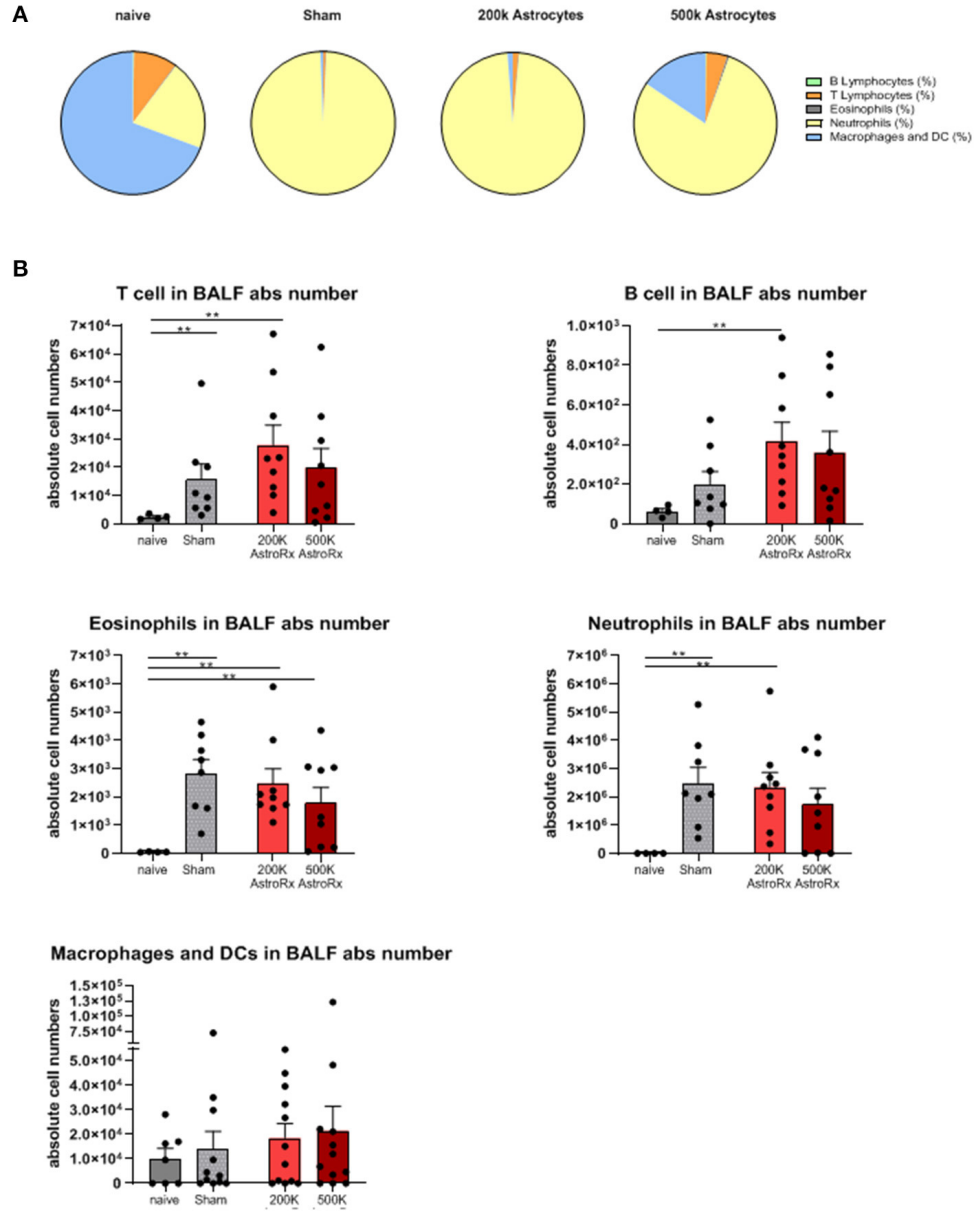
Immune cells analyses of bronchoalveolar lavage. Bronchoalveolar lavage (BALF) was analyzed by flow cytometry for T cells, B cells, eosinophils, neutrophils, and macrophages/dendritic cells. **(A)** Proportions of each cell population by treatment. **(B)** An absolute number of each cell population in the BALF. The results are expressed as mean ± SEM. The Mann–Whitney comparison test. Naïve vs. sham: **p = 0.0081, naïve vs. 200K astrocytes: **p = 0.0028 (T cells, eosinophils, and neutrophils), **p = 0.0056 (B cells), naïve vs. 500K astrocytes: **p = 0.0056 (eosinophils), **p < 0.01.

**Figure 4:**
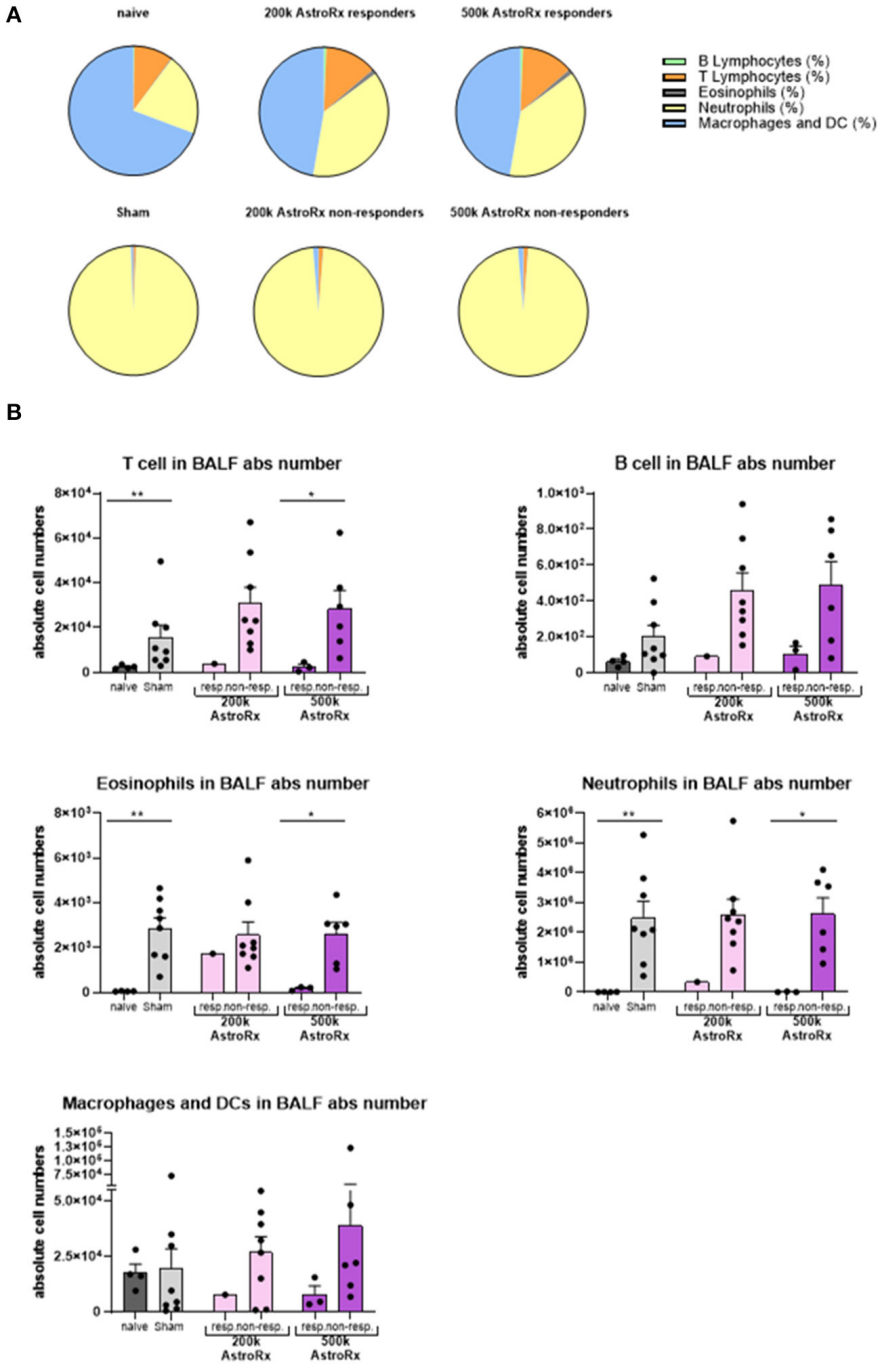
Responder mice maintain immune homeostasis in the lungs after AstroRx treatment. Bronchoalveolar lavage was analyzed by flow cytometry for the immune cells. **(A)** The composition of cells in the BALF of responders and non-responders after AstroRx treatment. **(B)** An absolute number of immune cells in the BALF. The value of *n* = 10 mice for each experimental group (sham and both the groups of AstroRx treated mice) and *n* = 4 for naïve mice, the results are expressed as mean ± SEM. The Mann–Whitney comparison test. *p < 0.05; **p < 0.01; Naïve vs. sham: **p = 0.0081 (T cells), **p = 0.0040 (eosinophils and neutrophils), 500k responders vs. non-responders: *p = 0.0286 (T cells, eosinophils, and neutrophils).

In the sham-injected ARDS mice, most of the cells in the BALF consisted of neutrophils (>92%), while the immune profile of high-dose of AstroRx shift toward naïve mice the immune profile (Figure 3, Supplementary Figure 1, *p* = 0.0274 for neutrophils and macrophages and *p* = 0.0206 for T cells). The effect of AstroRx on the immune cells became even clearer when we analyzed the immune profile differentially in the responder and non-responder mice (Figure 4, Supplementary Figure 2, *P* = 0.0286). The responder mice (lung score ≤2) among the AstroRx-treated LPS-injected mice had an immune profile that was similar to one of the naïve non-LPS-injected mice in terms of cell proportion and a general low-level of absolute cell number. While the non-responder mice displayed an immune profile that resembled the sham-injected mice with a general heavy infiltration of immune cells and a specifically massive invasion of neutrophils.

**Figure 5:**
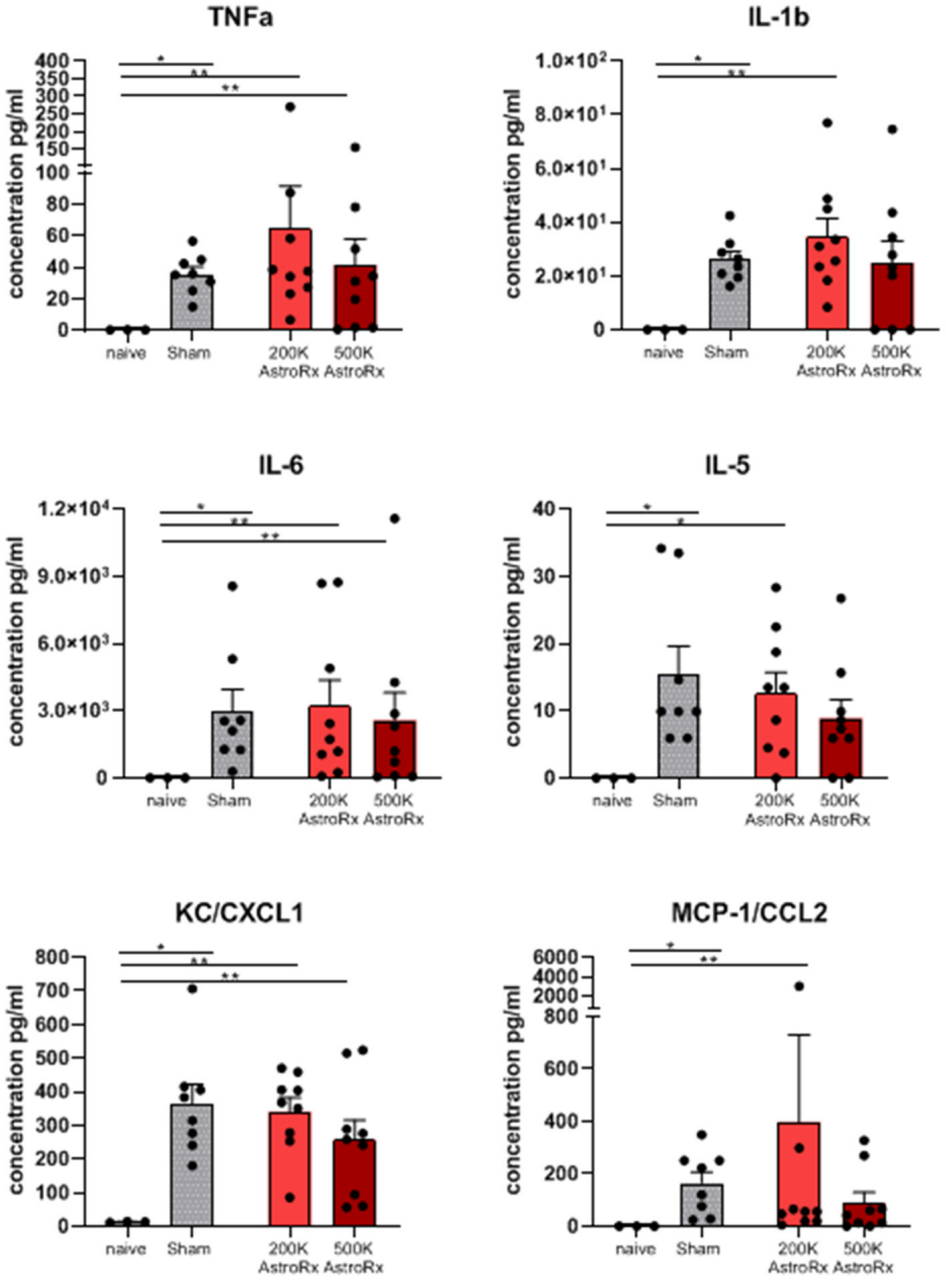
Inflammatory cytokines/chemokines in lungs. The cytokines and chemokines in the BALF were quantified by ELISA (enzyme linked immunosorbent assay). The value of *n* = 10 mice for each experimental group (sham and both the groups of AstroRx treated mice) and *n* = 4 for naïve mice, the results are expressed as mean ± SEM. The Mann–Whitney comparison test. *p < 0.05; **p < 0.01; Naïve vs. sham: *p = 0.0121, naïve vs. 200K or 500K astrocytes *p* = 0.0091 (TNFa, IL-6, CXCL1, and CCL2) and *p = 0.0318 (IL-5).

**Figure 6:**
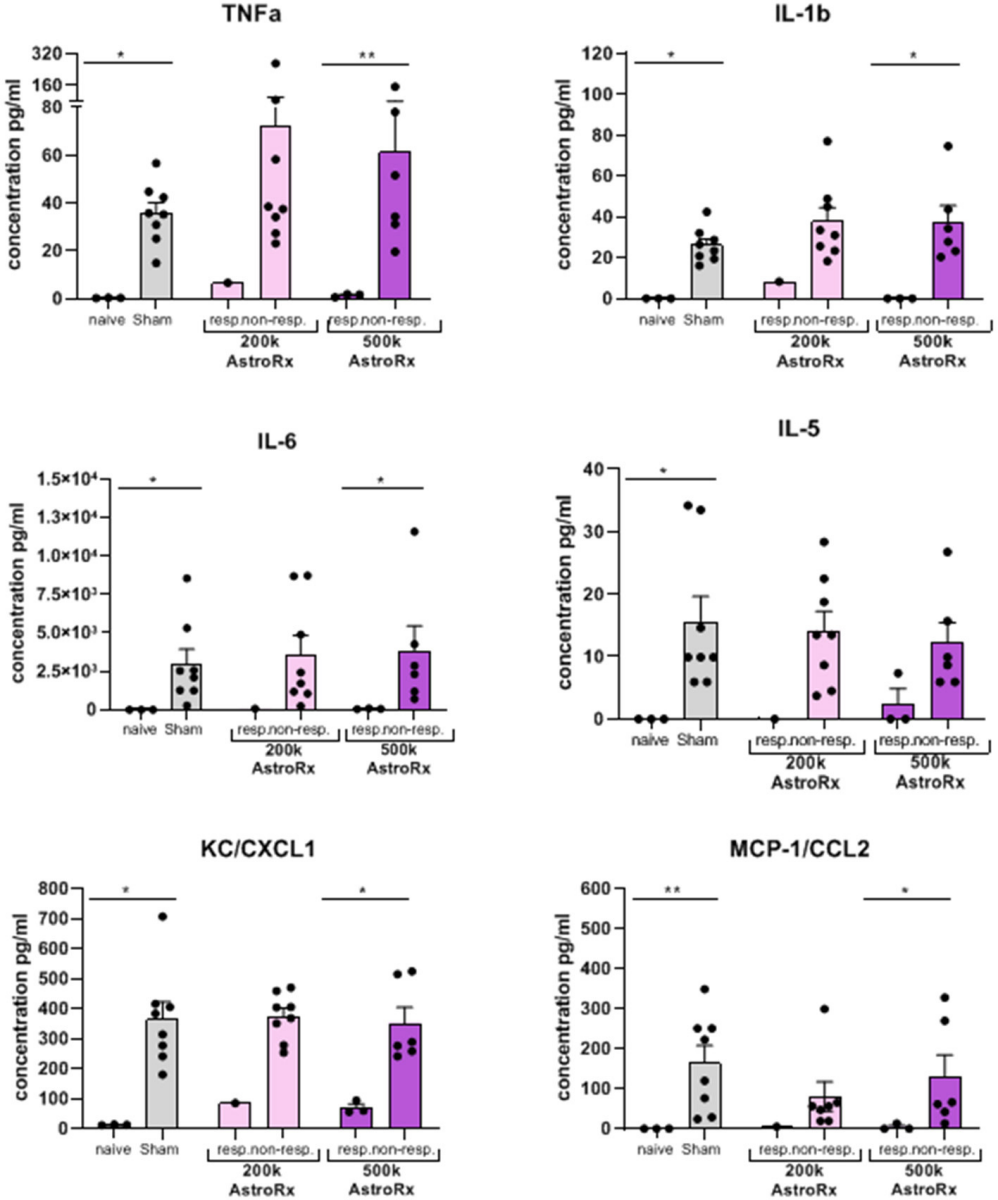
Inflammatory cytokines/chemokines in the lungs are held in check in responder mice treated with AstroRx cells. The cytokines and chemokines in the BALF were quantified by ELISA. The value of *n* = 10 mice for each experimental group (sham and both the groups of AstroRx treated mice) and *n* = 4 for naïve mice, the results are expressed as mean ± SEM. The Mann–Whitney comparison test. *p < 0.05; **p < 0.01; naïve vs. sham: *p = 0.0121 (for all cytokines/chemokines), 500K responders vs. non-responders: **p = 0.0091 (TNFa), *p = 0.0286 (IL-1b, IL-6, CXCL1, and CCL2).

As the immune profile is not only determined by the cells but also by molecules, we next analyzed the presence of cytokines and chemokines in the BALF and serum. At first sight, the expression of inflammatory cytokines and chemokines was quite heterogeneous within the group of AstroRx-treated animals (Figure 5, *P* = 0.13 and higher). But when we dissected the group again into responders and non-responders, it became clear, that the responders among the AstroRx treated mice showed low expression of TNFa, IL1b, IL-6, IL-5, CC2, and CXCL1 (Figure 6 naïve vs. sham: *P* = 0.0121 (for all cytokines); 500k responders vs. non-responders: *P* = 0.0091 (for TNFa), *P* = 0.0286 for all other cytokines). Strikingly, their levels were similar to the levels in naïve, healthy mice. At the same time, the non-responder mice displayed cytokine/chemokine levels in the range of sham-injected mice. The same phenomenon was observed in blood serum as well (Supplementary Figures 3, 4 naïve vs. sham: *P* = 0.0121 (for TNFa and CXCL1); 500k responders vs. non-responders *P* = 0.0235 [for TNFa and CXCL1)]. Altogether, our data showed that AstroRx had the capability to reduce the infiltration of immune cells to the lungs and to prevent the rising of a cytokine storm in the responder mice. It is reasonable to assume that this capability contributed to the better survival rate among the AstroRx-treated mice.

## Materials and Methods

### AstroRx Derived From Clinical Grade Human Embryonic Stem Cells (AstroRx®)

The protocol for manufacturing the human astrocytes (AstroRx®) from the embryonic stem cells was performed according to the protocol detailed in Izrael et al. (34). In brief, hESC line HADC100 was used as starting material for the derivation of AstroRx cells. The hESCs were grown in feeder free vitronectin coated flasks culture in essential 8™ (E8) medium (Thermo Fischer Scientific, MA, USA). Once enough cells were grown, hESC were detached to generate neurospheres (NS) in suspension (3D) cultures. The harvested hESC colonies were transferred into the 100-mm ultralow attachment culture plates (Corning) containing ITTSPP/B27 medium. The medium ITTSPP/B27 is a mixture of DMEM/F12 containing 1% B27 supplement, 1% glutamax, 1.5% HEPES at pH 7.4 (all from Thermo Fischer Scientific), 1% penicillin/streptomycin/amphotericin solution (Biological Industries, Israel), 25 μg/ml human insulin (ActRapid; Novo Nordisk, Denmark), 50 μg/ml human Apo-transferrin (Athens, GA, USA), 6.3 ng/ml progesterone, 10 μg/ml putrescine, 50 ng/ml sodium selenite, and 40 ng/ml triiodothyronine (T3) (all from Sigma, MO, USA). ITTSPP/B27 was supplemented with 20 ng/ml recombinant human epidermal growth factor (EGF) (R&D Systems, MN, USA). After 2 days, the medium was switched to ITTSPP/B27 supplemented with 20 ng/ml EGF and 10 μM all trans retinoic acid (Sigma, MO, USA). The culture was continued in suspension in the non-adherent plates for 7 days with daily replacement of the medium. During the last step, which allows for NS ripening, the culture was continued in ITTSPP/B27 medium supplemented with 20 ng/ml EGF for 18 days. The medium was replaced every other day. Then, round yellow NS were manually selected and transferred GMP-compliant laminin 521 (from Biolamina, Sweden) in ITTSPP/B27 supplemented with 20 ng/ml EGF. The medium was replaced every other day for 7–10 days (passage 0). To produce a monolayer of astrocyte progenitor cells (APC), the spheres were dissociated with TryplE (Thermo Fischer Scientific) and reseeded on laminin coated flasks in N2/B27 medium consisting of DMEM/F12 with 0.5% (v/v) N_2_ supplement, 1% (v/v) B27 supplement, 1% glutamax, and 1.5% HEPES (4-(2-hydroxyethyl)-1-piperazineethanesulfonic acid) at pH 7.4 (all from Thermo Fischer Scientific). The growth factors EGF and basic fibroblast growth factor (bFGF, R&D Systems) were added at 10 ng/ml each. The monolayer cells were further passaged weekly until enough cells were generated. The cells were then frozen in liquid nitrogen and stored as banks of APCs. Thawed APCs were further expanded and allowed to differentiate into the committed AstroRx cells by the removal of growth factors (EGF and bFGF), 50 μg/ml ascorbic acid (Sigma) was added, and the culture was continued for 7 days to yield AstroRx cells.

#### Animal Procedures

Female, 8 weeks old, BALB/C mice were obtained from Envigo (Israel) and maintained in SIA facility (Science in Action, Ness Ziona, Israel). Animal handling was performed according to the guidelines of the National Institute of Health (NIH) and the Association for Assessment and Accreditation of Laboratory Animal Care (AAALAC). The experiment was performed under the approval of “The Israel Board for Animal Experiments.” A total of 30 BALB/C female mice were anesthetized using isoflurane and treated through intratracheal route of administration with LPS (IT, 800 μg of LPS—ChemCruz, The Netherlands, 055:B5) to induce ARDS, the animals were randomized to receive 200K, 500K AstroRx cells, or PlasmaLyte (*n* = 10 mice per experimental arm). Naive mice (*n* = 4, without LPS instillation) were used as a control. Treated animals received i.v. injection of AstroRx cells or PlasmaLyte 6 h after LPS-induction. All the animals were sacrificed 72 h after the LPS instillation. The measurements of survival, body weight, hematology, lung histopathology, flow cytometry analysis of immune cells as well as cytokine and chemokines were collected from the BALF and serum. BALF was collected by intratracheal injection of 0.5 ml phosphate buffered saline (PBS) with 0.1 mM EDTA followed by a gentle aspiration three times. The recovered fluid was pooled and centrifuged. The BALF supernatant was preserved for the measurement of cytokines and chemokines. The sediment cells were resuspended and subjected to flow cytometry analysis.

#### Histology

Lungs were harvested and fixed in 4% formaldehyde. The tissues were then trimmed in a standard position and put in the embedding cassettes. One cassette per animal was prepared. The paraffin blocks were sectioned at ~ 4 μm thickness, put on the glass slides, and stained with H&E. Pictures were taken using an Olympus microscope (BX60, serial NO. 7D04032) at an objective magnification of × 4 and × 10 and microscope's Camera (Olympus DP73, serial NO. OH05504, Tokyo, Japan).

An analysis for ALI was performed according to the method described in Matute-Bello et al. using a severity scoring scale of 0–2, based on the American Thoracic Society Documents, 2011 (37). An analysis was performed by an independently certified veterinarian pathologist (Patho-logica Ltd., Ness Ziona, Israel) who was blinded to the experimental treatment.

##### Neutrophils

Not visible within the field—a score of 0; 1–5 neutrophils per field−1; more than 5 neutrophils per field−2.

##### Fibrin

Not visible within the field—a score of 0; a single well-formed band of fibrin within the airspace−1; multiple eosinophilic membranes−2.

##### Thickened Alveolar Walls

Due to technical artifacts, only septal thickening that is ≤ two times the normal was considered. Less than ×2—score 0; ×2– ×4—score 1; more than × 4—score 2.

The analysis was based on the measurements of 20 fields, using an objective magnification of ×4 and ×10 (high power fields, HPF).

Neutrophil cell count was performed using MATLAB color-based, brightness-based, and morphological-based segmentation. The cells were counted from a rectangle of 88,892 μm^2^.

#### Cytokine and Chemokine Multiplex Measurements

The BALF cytokine concentrations were measured using the ProcartaPlex Luminex platform (Thermo Fischer Scientific, MA, USA). The measurements were performed two times (25 μl of each sample) with a custom multiplex panel detecting the following mouse cytokines: IFNγ, TNFα, RANTES, IL-6, IL-10, IL-1α, IL-1β, IP-10, MIP1α, and MCP-1/CCL2. The measurements were performed using the Luminex MAGPIX instrument, and results were analyzed with Xponent 4.2 software according to the instructions from the manufacturer.

##### Mixed lymphocyte Reaction Test

Three female C57/Bl6 mice at age of 6–8 weeks were euthanized by injection of a lethal dose of Pental (Pentobarbital Sodium). The lymph nodes were excised and drained to obtain lymph-node cells (LNCs). The LNCs from all the mice were pooled and then counted by a hemocytometer using trypan blue dye to exclude the dead cells. One million LNCs were seeded in RPMI-1640 medium (Biological Industries, 01-100-1A) supplemented with 2.5% fetal calf serum (Biological Industries, 04-121-1A), 1 mM L-glutamine (Biological Industries, 03-020-1C), and penicillin-streptomycin (Biological Industries, 03-031-1B). The LNC cultures under the multiple experimental conditions were maintained in flat-bottom plates, in duplicates, in a humidified atmosphere of 5% carbon dioxide at 37°C. The mNPCs were obtained using the method described in Fainstein and Ben-Hur (35) for isolation and growth of mNPCs. The mNPCs were kept in culture with the same media used to culture LNCs. The cells were harvested and analyzed for T-cell amount and proliferation by flow cytometry. Analysis of cells that expressed both Thy1.2 and CD25 determined the percentage of T cells in LNC culture, 24 h after activation with ConA.

### BrdU T-cell Proliferation Assay

Co-expression of CD3/BrdU determined the percentage of proliferating T cells in co-cultures with LNC. The LNCs were kept in culture with and without 2.5 μg/ml ConA (Sigma, C5275) for 48 h. The percentage of T cells (Thy1.2^+^/CD25^+^) and proliferating T cells (CD3^+^/BrdU^+^) of the LNC cultures in the presence of ConA were set as baselines for the T-cell activation—without immunomodulation. The AstroRx® cells were added to LNC culture at ratios of 2:1, 5:1, and 10:1 LNC:AstroRx®, with and without 2.5 μg/ml ConA. Further, the LNCs in 0.5 ml RPMI-1640 were cultured with 0.5 ml conditioned medium with and without 2.5 μg/ml ConA. As a positive control, the mNPCs were added to the LNC culture at a ratio of 2:1 with and without 2.5 μg/ml ConA.

### Flow Cytometry

The cells were analyzed by flow cytometry for identity and purity markers using the following antibodies: anti-GLAST (1:20; Miltenibiotec, Germany), anti-CD44 (1:20; BD Pharmingen, CA, USA), anti-CXCR4 (1:20; Biolegend, CA, USA), anti-TRA-1-60 (1:50; Biolegend), anti-EPCAM (1:50; Biolegend), anti-SSEA4 (1:50; Biolegend), anti-GFAP (1:2000; Sigma), anti-Nestin (1:500; BD Pharmingen), and anti-AQP-4 (1:2000; Abcam, UK). The Flow Cytometer FACS Canto II (BD, NJ, USA) was operated with FACSDIVA software (BD). At least 10,000 events were collected per sample. For immune cells identity, the following antibodies were used: FITC Rat Anti-Mouse I-A/I-E Clone 2G9 (RUO) (BD 553623), PerCP-Cy™5.5 Hamster Anti-Mouse CD3e (BD 551163), PerCP-Cy™5.5 Rat Anti-Mouse CD45R/B220 (552771), Mouse CCR3 PE-conjugated Antibody (R&D FAB729P), and APC-anti-mouse CD11 (Biolegend BLG-117310d).

### Statistical Analyses

The statistical analyses were performed using GraphPad Prism 7 software (GraphPad Software, San Diego, CA, USA). The *p*-values were calculated by Mann–Whitney test. The values ≤0.05 were considered significant (^*^ *p* < 0.05; ^**^ *p* < 0.01; and ^***^ *p* < 0.001).

## Discussion

We are currently evaluating the curative role of AstroRx in patients with ALS. In a Phase I/IIa, open-label, dose-escalating clinical study, we studied the safety, tolerability, and therapeutic effects of AstroRx intrathecal transplantation.

Similarly to ARDS, the levels of circulating chemokines and cytokines in ALS are increased. The patients with a shorter diagnostic delay, which is a marker of more severe rapidly progressing disease (38) display higher levels of the inflammatory chemokine MCP-1/CCL-2. In addition, the increased levels of the inflammatory cytokines IL-17 and IL-6 were detected in the serum of the patients with ALS (39, 40). The increased levels of LPS in patients with ALS suggest systemic inflammation (41).

In preclinical studies, the AstroRx cells demonstrated a neuron-protective phenotype (A2 astrocytes) and secreted neurotrophic factors that were capable to uptake glutamate and regulate oxidative stress (34). Depending on their mode of activation (A1/A0/A2) (23, 25), astrocytes may possibly play an anti-inflammatory (A2) or pro-inflammatory (A1) role.

To test the immunomodulatory effect of AstroRx cells, we chose a rapid and acute inflammation model. LPS lung instillation is one of the most used models for ARDS. This model shares a number of pathological features with COVID-19-related ARDS, such as hypoxemia, neutrophil accumulation, alveolar space thickening, fibrin and tissue pathology, and high levels of inflammatory cytokines (2, 10, 13). The similarities between the LPS-treated rodents and the patients with COVID-19, in terms of lung damage and the inflammatory response, make LPS-induced ARDS a reliable model to evaluate potential COVID-19 therapies.

Administration of cells, mainly MSC has been performed by either local or systemic routes in the different experimental models (42). Local administration via intratracheal or intrathoracic infusion delivers the cells directly to the site of injury, whereas systemic administration *via* intravenous infusion allows wide distribution throughout the body. The cells administered intravenously would encounter the first-class pulmonary effect (43), which might result in the significant retention of cells in the lung, thereby providing advantages for lung tissue repair. The ongoing clinical trials and most experimental studies have used intravenous route for MSC administration (44, 45). Therefore, intravenous infusion of AstroRx cells was selected as a route of administration for testing ARDS.

In the current study, the levels of TNF-alpha, IL-b1, and IL-6 were strongly elevated in LPS-induced animals but could be maintained in steady state in the responder mice of AstroRx and LPS-treated group. The expression of CCL2, a monocyte recruiter, and CXCL1, a neutrophil recruiter, was also increased in the LPS-induced sham animals, and significantly reduced in the AstroRx responder mice. The expression level of IL-6, IL1b, TNFα, and CXCL1 determined if the lung remained in a homeostatic state or became inflamed. The upregulated levels of CXCL1 and TNF were also detected in the peripheral blood. Astrocytes calcium signaling is implicated in regulating inflammation (46–49). Similar to the pathology reported in ALS disease, the levels of pro-inflammatory cytokines, such as TGF-β1, IL-10, IFNγ, and IL-6 are elevated in LPS-induced ARDS animals. Exposure of astrocytes to inflammatory stimuli (e.g., TGF-β1 and IFNγ) demonstrated that inflammatory stimuli can significantly alter the astrocyte calcium signaling elicited by multiple G-protein-coupled receptors (GPCRs) (50). Alterations in the GPCR-evoked astrocyte Ca^2+^ transients may represent mechanisms by which astrocytes regulate inflammation (51) and should be further investigated.

Our current study demonstrated the immunosuppressive capacity of AstroRx cells in the context of lung inflammation and presents AstroRx as an innovative approach for treating ARDS especially in light of the rising unmet need for a treatment for COVID-19-related ARDS.

To make AstroRx cell therapy clinically applicable, further research is needed to elucidate several issues, such as the optimal number of AstroRx cell doses, the time window of AstroRx cell administration, administration routes, and frequency (single vs. multiple-dose regimen). Comprehensive safety investigation on the fate of AstroRx cells was done once the cells were injected intrathecally into the cerebrospinal fluid (34). The safety profile of AstroRx cells upon i.v. injection requires further investigation in the terms of cell survival, homing capacity to different tissues (e.g., lungs, liver, and kidney), cell identity, and reactive state (A1/A2) after cell transplantation.

## Data Availability Statement

The original contributions presented in the study are included in the article/supplementary material. Further inquiries can be directed to the corresponding author.

## Ethics Statement

The animal study was performed according to guidelines of the National Institute of Health (NIH) and the Association for Assessment and Accreditation of Laboratory Animal Care (AAALAC). The experiment was performed under the approval of The Israel Board for Animal Experiments.

## Author Contributions

MR, MI, AR, and JMW conceived and designed the studies. MI, JMW, SGS, TS, and KM performed the experiments, analyzed the data, and interpreted the data. MR, MI, and JMW wrote the manuscript. All authors read and approved the final manuscript.

## Conflict of Interest

MI, AR, SGS, KM, TS, JMW, and MR are employed by company Kadimastem Ltd.

## Publisher's Note

All claims expressed in this article are solely those of the authors and do not necessarily represent those of their affiliated organizations, or those of the publisher, the editors and the reviewers. Any product that may be evaluated in this article, or claim that may be made by its manufacturer, is not guaranteed or endorsed by the publisher.

## Supplementary Material

Supplementary Figure 1 The percentage analyses of immune cells in bronchoalveolar lavage. Bronchoalveolar lavage (BALF) of mice was analyzed by flow cytometry for lymphocytes and myeloid cells. The results are expressed as mean ± SEM. Mann–Whitney comparison test. ^*^*p* < 0.05; ^**^*p* < 0.01. Sham (*n* = 10) vs. 200k astrocytes treated mice (*n* = 10): ^*^*p* = 0.0152 (T cells), sham vs. 500k astrocytes treated mice (*n* = 10): ^*^*p* = 0.0206 (T cells), sham vs. 500k astrocytes treated mice: ^*^*p* = 0.0274 (neutrophils and eosinophils).

Supplementary Figure 2 Immune cell distribution in the lungs resembles naïve, healthy mice. The BALF of mice was analyzed by flow cytometry for the percentage of lymphocytes and myeloid cells divided into responder and non-responders mice. The results are expressed as mean ± SEM. Mann–Whitney comparison test. ^*^*p* < 0.05; ^**^*p* < 0.01; Naïve vs. sham: ^**^*p* = 0.0040 (T cells, neutrophils, and macrophages), ^**^*p* = 0.0028 (B cells). 500k responders vs. non-responders: ^*^*p* = 0. 0286 (all cell types).

Supplementary Figure 3 CXCL1 and TNFa levels in the peripheral blood of AstroRx-cell treated mice mirror the situation in the lungs. CXCL1 and TNFa levels in the blood serum were quantified by ELISA. *n* = 10 mice for each experimental group [sham and both groups of human astrocytes (AstroRx) treated mice] and *n* = 4 for naïve mice, results are expressed as mean ± SEM. Mann–Whitney comparison test. ^*^*p* < 0.05; ^**^*p* < 0.01; ^***^*p* < 0.001.

Supplementary Figure 4 The CXCL1 and TNFa levels in the peripheral blood of AstroRx-cell treated mice mirror the situation in the lungs. The CXCL1 and TNFa levels divided by responder and non-responder mice in the blood serum were quantified by ELISA. The results are expressed as mean ± SEM. Mann–Whitney comparison test. ^*^*p* < 0.05.
