# Supplementary figures and images for: Astrocytes Downregulate Inflammation in Lipopolysaccharide-Induced Acute Respiratory Distress Syndrome: Applicability to COVID-19
Source: Front Med (Lausanne). 2021 Oct 29;8:740071. doi: 10.3389/fmed.2021.740071 (PMC8585990; doi:10.3389/fmed.2021.740071)

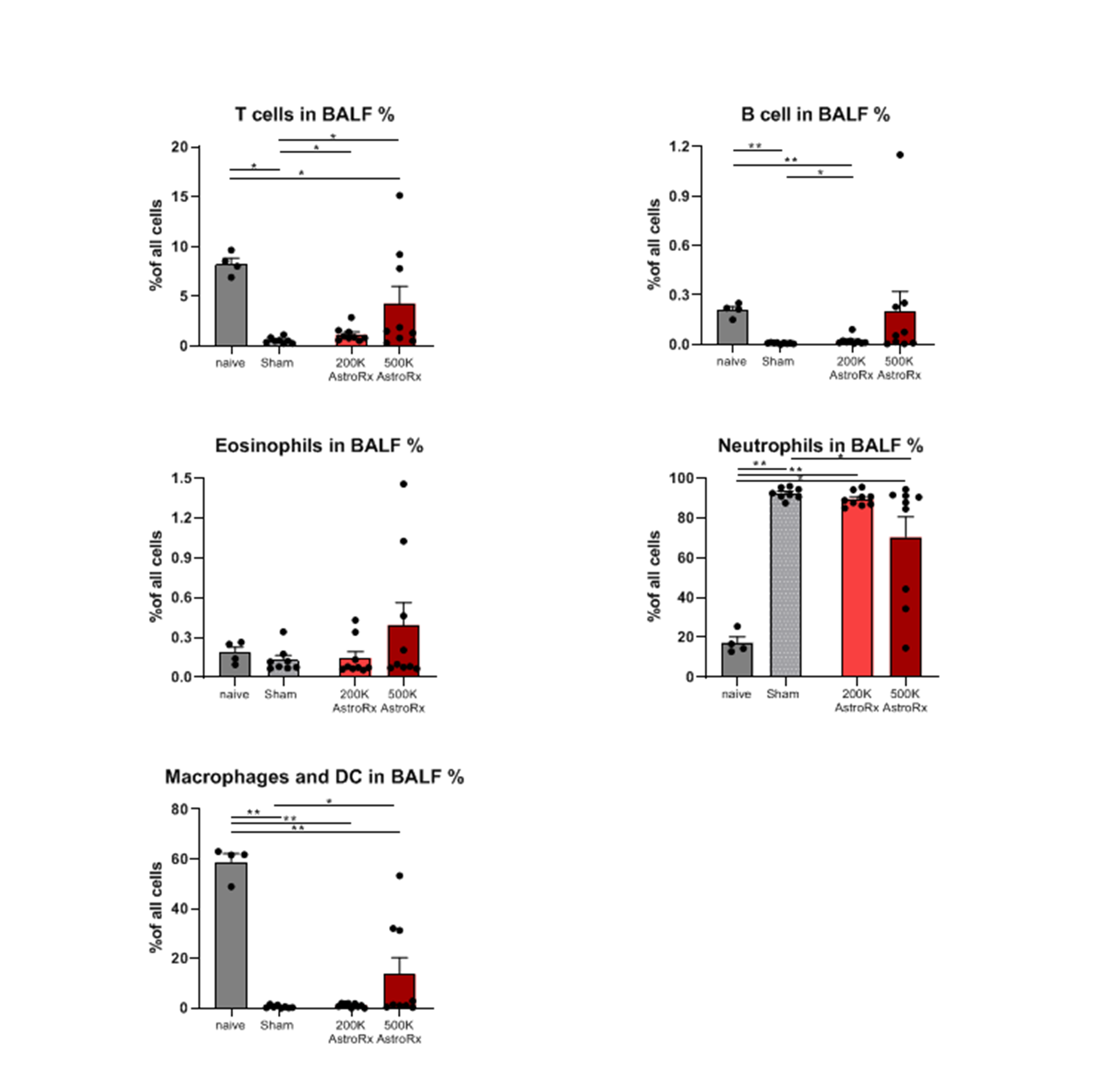

Supplement: Supplementary Figure 1 — The percentage analyses of immune cells in bronchoalveolar lavage. Bronchoalveolar lavage (BALF) of mice was analyzed by flow cytometry for lymphocytes and myeloid cells. The results are expressed as mean ± SEM. Mann–Whitney comparison test. *p < 0.05; **p < 0.01. Sham (n = 10) vs. 200k astrocytes treated mice (n = 10): *p = 0.0152 (T cells), sham vs. 500k astrocytes treated mice (n = 10): *p = 0.0206 (T cells), sham vs. 500k astrocytes treated mice: *p = 0.0274 (neutrophils and eosinophils). [file Image_1.TIF]

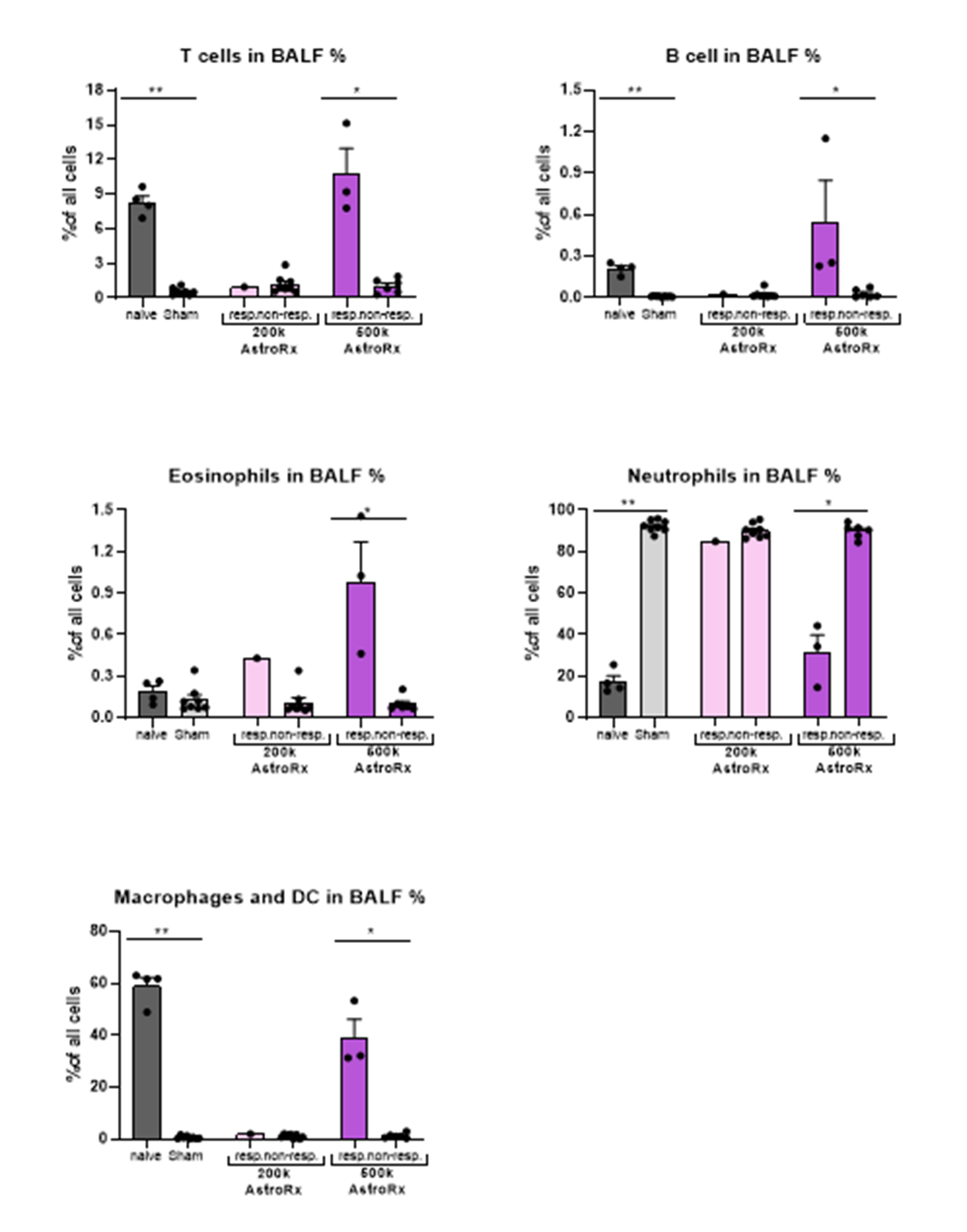

Supplement: Supplementary Figure 2 — Immune cell distribution in the lungs resembles naïve, healthy mice. The BALF of mice was analyzed by flow cytometry for the percentage of lymphocytes and myeloid cells divided into responder and non-responders mice. The results are expressed as mean ± SEM. Mann–Whitney comparison test. *p < 0.05; **p < 0.01; Naïve vs. sham: **p = 0.0040 (T cells, neutrophils, and macrophages), **p = 0.0028 (B cells). 500k responders vs. non-responders: *p = 0. 0286 (all cell types). [file Image_2.TIF]

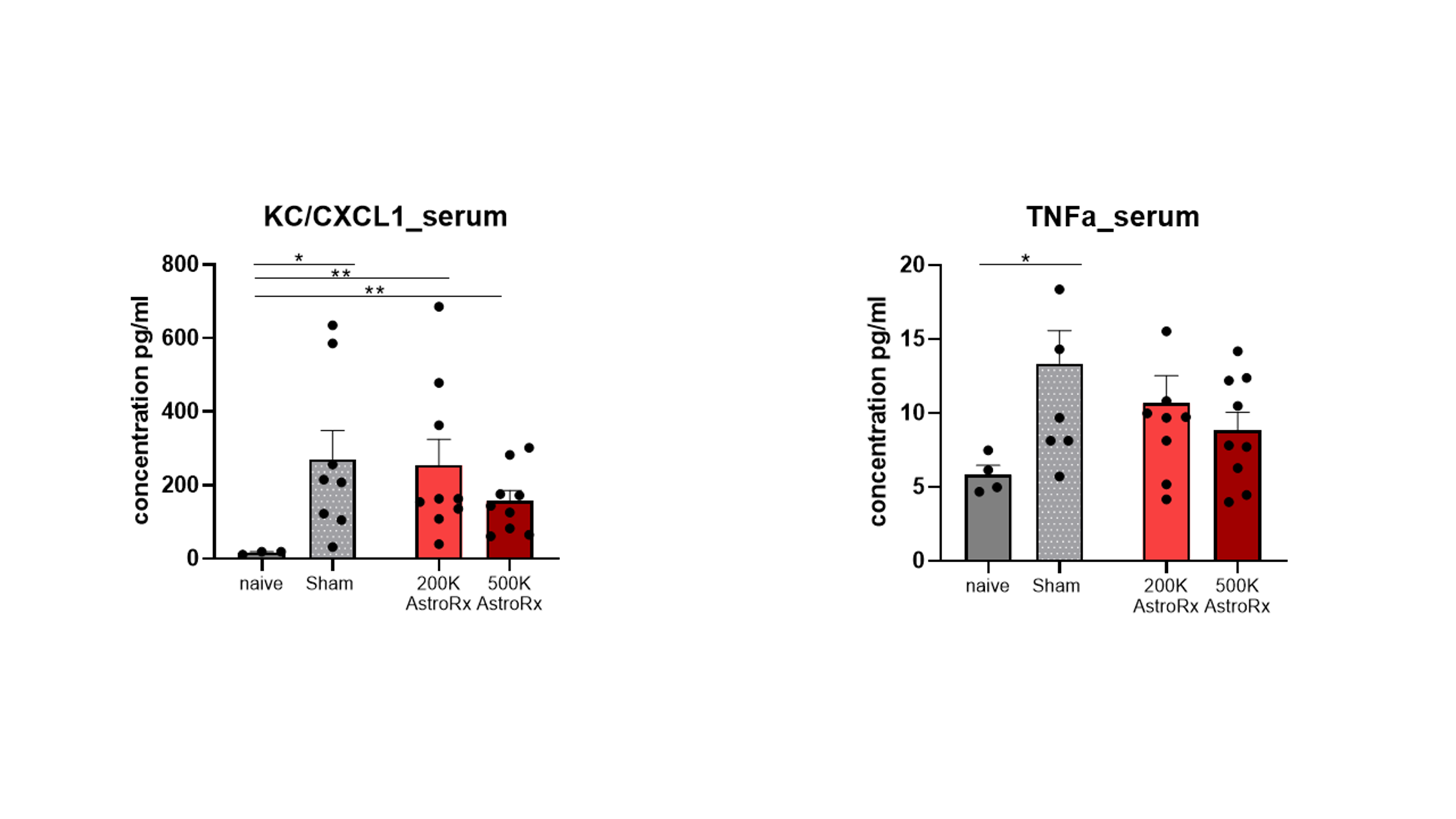

Supplement: Supplementary Figure 3 — CXCL1 and TNFa levels in the peripheral blood of AstroRx-cell treated mice mirror the situation in the lungs. CXCL1 and TNFa levels in the blood serum were quantified by ELISA. n = 10 mice for each experimental group [sham and both groups of human astrocytes (AstroRx) treated mice] and n = 4 for naïve mice, results are expressed as mean ± SEM. Mann–Whitney comparison test. *p < 0.05; **p < 0.01; ***p < 0.001. [file Image_3.TIF]

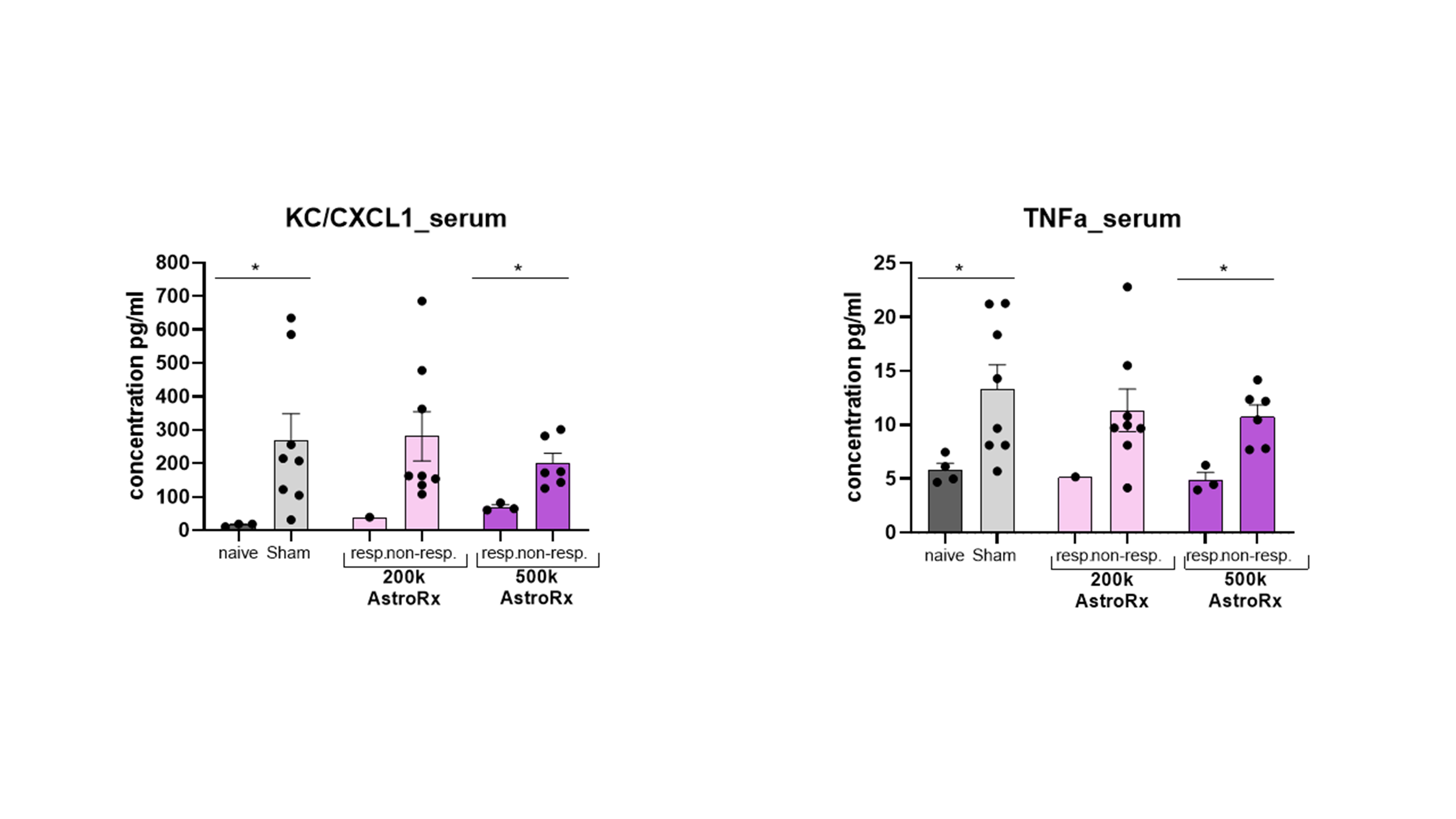

Supplement: Supplementary Figure 4 — The CXCL1 and TNFa levels in the peripheral blood of AstroRx-cell treated mice mirror the situation in the lungs. The CXCL1 and TNFa levels divided by responder and non-responder mice in the blood serum were quantified by ELISA. The results are expressed as mean ± SEM. Mann–Whitney comparison test. *p < 0.05. [file Image_4.TIF]
